# High Prevalence of New Delhi Metallo-β-Lactamase-1 (NDM-1) Producers among Carbapenem-Resistant *Enterobacteriaceae* in Kuwait

**DOI:** 10.1371/journal.pone.0152638

**Published:** 2016-03-31

**Authors:** Wafaa Y. Jamal, M. John Albert, Vincent O. Rotimi

**Affiliations:** Department of Microbiology, Faculty of Medicine, Kuwait University and Microbiology Unit, Mubarak Al Kabir Hospital, Jabriya, Kuwait; Second University of Naples, ITALY

## Abstract

The aim of the study was to determine the prevalence of New Delhi metallo-β lactamase-1 (NDM-1) producing *Enterobacteriaceae* in Kuwait over a one year period. Consecutive *Enterobacteriaceae* isolates with reduced susceptibility to carbapenems were collected from four government hospitals in Kuwait from January–December 2014. Their susceptibility to 18 antibiotics was performed by determining the minimum inhibitory concentration. Isolates resistant to carbapenems were tested by PCR for resistant genes. Finger printing of the positive isolates was done by DiversiLab^®^. Clinical data of patients harboring NDM-1 positive isolates were analyzed. A total of 764 clinically significant *Enterobacteriaceae* isolates were studied. Of these, 61 (8%) were carbapenem-resistant. Twenty one out of these 61 (34.4%) were NDM-1-producers. All patients positive for NDM-1-carrying bacteria were hospitalized. About half were females (11/21 [52.3%]), average age was 53.3 years and the majority were Kuwaitis (14/21 [66.6%]). Six patients (28.5%) gave a history of travel or healthcare contact in an endemic area. Mortality rate was relatively high (28.6%). The predominant organism was *Klebsiella pneumoniae* (14 [66.6%]) followed by *E*. *coli* (4 [19%]). All NDM-1-positive isolates were resistant to meropenem, ertapenem, cefotaxime, cefoxitin and ampicillin, while 95.2% were resistant to imipenem, cefepime, and piperacillin-tazobactam. They were multidrug resistant including resistance to tigecycline, but 90% remained susceptible to colistin. About two-thirds of isolates (61.9%) co-produced-extended spectrum β-lactamases. During the study period, an outbreak of NDM-1 positive *K*. *pneumoniae* occurred in one hospital involving 3 patients confirmed by DiversiLab^®^ analysis. In conclusion, NDM-1-producing *Enterobacteriaceae* is a growing healthcare problem with increasing prevalence in Kuwait, especially in hospitalized patients, leaving few therapeutic options. A high prevalence of NDM-1 necessitates the implementation of strict infection control to prevent the spread of these organisms.

## Introduction

Infections due to *Enterobacteriaceae* are an important cause of morbidity and mortality worldwide. Use of antimicrobial agents is the main stay of treatment of such infections. However, resistance to these agents has become a serious health concern worldwide [1]. Carbapenems, e.g. imipenem and meropenem, are the last choice to treat multidrug- resistant (MDR) Gram-negative bacterial infections [2]. Infections due to carbapenem-resistant *Enterobacteriaceae* (CRE) are increasingly reported throughout the world and the Centers for Disease Control and Prevention (CDC), USA considers them as an urgent threat to human health [3]. Carbapenem resistance in *Enterobacteriaceae* can be mediated by production of carbapenemases which are encoded by different genes [2]. Carbapenemases in *Enterobacteriaceae* can be divided into Ambler class A β- lactamases (e.g. KPC), class B metallo-β-lactamases (MBLs) (e.g. VIMs, IMPs, NDMs) and class D oxacillinases (e.g. OXA-48 like enzymes) [2]. New Delhi metallo-β-lactamase-1 (NDM-1), a relatively newly described MBL, can hydrolyze all β-lactams including carbapenems except monobactam. It was first identified in *Klebsiella pneumoniae* and *Escherichia coli* isolated from a Swedish patient who was hospitalized in India in 2008 [4]. Since then, it has spread all over the world. To-date, NDM-1-producing-bacteria have been found in over 40 countries creating a significant public health threat [1, 5, 6]. NDM-1 producing bacteria are particularly important because the gene encoding this enzyme is found on transferable plasmid (of varying size) so that resistance can be easily transferred from one bacterium to another. These bacteria are usually multi-drug resistant making treatment difficult. NDM-1-positive isolates are also colonizers, causing no disease and therefore screening for them can be difficult. Importantly, NDM-1 enzyme is also present in *E*. *coli*, the most common cause of urinary tract infection in the community and hospital [7, 8].

Kuwait is a Middle-Eastern country of 3.5 million people, the majority of whom are expatriates from numerous countries. This population mix provides ample opportunities for introduction and spread of resistant bacteria. In Kuwait, NDM-1 producing *K*. *pneumoniae* was first identified in two hospitalized patients [9]. A subsequent study in a major teaching hospital found that *K*. *pneumoniae* was the main species that expresses NDM-1 [10]. However, the magnitude of carbapenem resistance due to NDM-1 in *Enterobacteriaceae* in Kuwait is unknown. We therefore, conducted a surveillance study to detect carbapenem-resistant *Enterobacteriaceae* carrying NDM-1 gene in four major hospitals in Kuwait over a one—year period.

## Materials and Methods

A surveillance study was conducted in four major teaching/public hospitals in Kuwait (Amiri, Farwaniya, Ibn Sina, and Mubarak Al Kabeer hospitals) responsible for healthcare delivery to a catchment area of 2.5 million people. Consecutive clinically significant (suspected of causing infection or carriage) isolates of the family *Enterobacteriaceae* were collected from a total of 764 patients during one year, January to December 2014. These were non-duplicate single isolate per patient. The contribution from each hospital was as follows: Amiri hospital, 208; Farwaniya hospital, 123; Ibn Sina hospital, 210; and Mubarak Al Kabeer hospital, 223. The isolates were from different sources e.g. urine, wound and tissue, blood, respiratory secretion, abscess, central venous pressure line tip, peritoneal dialysis fluid, cerebrospinal fluid, pericardial fluid and screening rectal and throat swabs.

### Bacterial isolates

A total of 61 non-susceptible isolates to at least one of the carbapenems, with a minimum inhibitory concentration (MIC) of >1mg/L for imipenem or meropenem, or >0.5mg/L for ertapenem, according to the Clinical and Laboratory Standards Institute (CLSI) [11] were included in the study. They were stored at -70°C in 1% protease/peptone broth containing 7% glycerol for further evaluation. Species identification was performed with VITEK II automated system (bioMérieux, Marcy l’Etoile, France).

### Antimicrobial susceptibility testing and phenotypic assays

Antibiotic susceptibility testing was performed using E test (bioMérieux). The tested antibiotics were: amikacin and gentamicin (aminoglycosides); ampicillin, amoxicillin-clavulanic acid and piperacillin-tazobactam (β-lactams); aztreonam (monobactam); cephalothin, cefepime, cefotaxime, cefoxitin, ceftazidime, and cefuroxime (cephalosporins); ciprofloxacin (quinolone); colistin (polymyxin); ertapenem, imipenem and meropenem (carbapenems) and tigecycline (glycylcycline). Susceptibility testing was interpreted according to the Clinical Laboratory Standards Institute (CLSI) recommendations [11], except for tigecycline and colistin. For tigecycline, we used the breakpoints recommended by the Food and Drug Administration, USA (i.e. susceptible if MIC was ≤ 2mg/L and resistant if MIC was ≥ 8mg/L). For colistin, breakpoints recommended for *Acinetobacter* spp. was used (i.e. MIC of ≤ 2mg/L for susceptibility and MIC of ≥ 4mg/L for resistance) [11]. *E*. *coli* ATCC 25922 and *E*. *coli* ATCC 35218 strains were used as controls. Initial screening for the presence of carbapenemase was done by the modified Hodge test (MHT) [11] and E test. *K*. *pneumoniae* ATCC BAA-1705 and *K*. *pneumoniae* ATCC BAA-1706 were used as positive and negative controls, respectively.

### PCR amplifications and sequencing

PCR assays were used to amplify the extended-spectrum β-lactamases (ESBLs) genes- *bla*_CTX-M_, *bla*_SHV_ and *bla*_TEM_, [12] and the carbapenemase genes- *bla*_OXA-48_, *bla*_VIM_, *bla*_NDM_, *bla*_IMP_, *bla*_GIM_ and *bla*_KPC_ [13]. Sequencing of the amplicons was performed using a GenAmp PCR system 9700 by cycle sequencing with BigDye terminator (AB Applied Biosystem, Carlsbad, California, USA).

### Molecular finger printing

Clonal relatedness of the *K*. *pneumoniae* and *E*. *coli* isolates was investigated by semi-automated repetitive sequence-based polymerase chain reaction (rep-PCR) using the DiversiLab^®^ strain typing platform (bioMérieux). It was performed in duplicate with the DiversiLab^®^ *Klebsiella* spp. kit and *Escherichia* spp. kit, according to manufacturer’s instructions. The resulting electropherograms were analyzed with DiversiLab software version 3.4 (bioMérieux). Samples were compared by automatically generated dendrograms calculated using the unweighted pair- group method with arithmetic mean (UPGMA) algorithm. In DiversiLab^®^, the cut-off limits for identity, similarity and dissimilarity for both *E*. *coli* and *K*. *pneumoniae* are ≥ 98%, ≥ 95% and < 95% respectively, [14, 15].

### Ethics statement

Collection of the strains was conducted according to the Declaration of Helsinki and with particular institutional ethical and professional standards. A written informed consent was not obtained from patients or parents of children because the bacterial isolates studied were collected from the routine work of clinical microbiology laboratory for patient care and no additional clinical specimens were collected for the purpose of the study. It is a standard practice not to get written informed consent for use of bacterial isolates unlinked to patient identity from the routine clinical laboratory. Therefore, the waiver for informed consent was granted and the study was approved by the Medical Ethics Committee of Ministry of Health, Kuwait approved the study (permit number 288/MTT).

## Results

### Bacterial isolates

Sixty one (8%) of 764 *Enterobacteriaceae* isolates collected were carbapenem- resistant *Enterobacteriaceae* (CRE) made up of *K*. *pneumoniae* (n = 25), *E*. *coli* (n = 22), *Morganella morganii* (n = 6), *Enterobacter cloacae* (n = 5), *Enterobacter aerogenes* (n = 2), and *Providencia stuartii* (n = 1). Of these 61 CRE, 21 (34.4%) were positive for *bla*_NDM-1_ and belonged to different bacterial species: *K*. *pneumoniae* (n = 14; 66.6%), *E*. *coli* (n = 4; 19%), *E*. *cloacae* (n = 1; 4.7%), *M*. *morganii* (n = 1; 4.7%) and *P*. *stuartii* (n = 1; 4.7%). The clinical features and characteristics of these 21 patients are shown in Table 1. The age of the patients ranged from 5 months to 83 years (mean = 53.3 years). Male—to—female ratio was 10:11 (0.9). The majority were Kuwaitis (n = 14; 66.6%) followed by Indians (n = 4; 19%) and one each from Egypt, Iran and Syria (4.8%). They were all residents of Kuwait. All the patients with NDM-1 positive isolates were infected except two patients who were colonized in the rectum, and throat (patient numbers 9 and 15, respectively). Before development of CRE infection, patients received prior antimicrobial therapies numbering from one to 7 antimicrobial agents. Fifteen (71.4%) patients were discharged alive from the hospital, while all 6 (28.6%) patients with sepsis and septic shock died.

**Table 1:** Clinical features and patient characteristics associated with carbapenem-resistant *Enterobacteriaceae* isolates producing NDM-1.

Patient	Age/ gender	Nationality	Underlying disease	Organism	Antibiotic therapy	Outcome
1	83y/male	Kuwaiti	Chest infection, Right hip replacement	*E*. *coli*	TZP, CLAR	Survived
2	52y/male	Indian	Bladder cancer, DM, BP	*Morganella morganii*	AMC, CIP, NIT, CST	Survived
3	82y/female	Egyptian	CVA, BP, epilepsy, aspiration pneumonia, infected bed sore	*K*. *pneumoniae*	FEP, MTZ, MEM, TGC, CST	Died from sepsis
4	53y/male	Indian	Bilateral urolithiasis	*E*. *coli*	AMK, CRO	Survived
5	80y/female	Kuwaiti	CVA, DM, CKD on dialysis, DVT, PVD	*K*. *pneumoniae*	CIP, AMK,	Survived
6	78y/female	Kuwaiti	DM, IHD, obstructive uropathy, CKD	*K*. *pneumoniae*	MEM, AMB, CIP, VAN	Survived
7	66y/male	Kuwaiti	DM, BP, CVA aspiration pneumonia, CVA	*Providencia stuartii*	MEM, CIP, TZP, CRO	Died from sepsis
8	1y/female	Indian	Atypical familial uremic syndrome	*K*. *pneumoniae*	AMC, GEN	Survived
9	5month/female	Kuwaiti	Left sided hip dislocation, bronchial asthma, pneumonia	*E*. *coli*	CTX	Survived
10	56y/male	Syrian	Bilateral obstructive uropathy, BP, kidney stones	*Enterobacter cloacae*	CRO	Survived
11	67y/female	Kuwaiti	DM, BP, old CVA, IHD, bronchial asthma, thyroidectomy	*K*. *pneumoniae*	TZP, CLAR, MEM, CAZ, CST	Died from sepsis
12	68y/female	Kuwaiti	BP, DM, atrial fibrillation, CVA	*K*. *pneumoniae*	TZP, CAZ, CRO, MEM, FEP, TEC FLC	Survived
13	60y/female	Kuwaiti	DM, BP, mixed connective tissue disease, pneumonia	*K*. *pneumoniae*	TZP, MTZ	Survived
14	69y/female	Kuwaiti	CML, DM, BP	*K*. *pneumoniae*	TZP, VAN, AMK, MEM, FLC	Died from sepsis
15	34y/male	Iranian	Drug overdose, cardiac arrest, hypoxic encephalopathy, aspiration pneumonia	*K*. *pneumoniae*	CRO, MTZ, TZP, IPM, MEM, TGC, CST	Survived
16	36y/female	Kuwaiti	SLE, lupus nephritis, hereditary elliptocytosis	*E*. *coli*	FLC	Survived
17	48y/male	Indian	DM, BP, CVA	*K*. *pneumoniae*	CRO, CLAR, MEM, CST	Died from sepsis
18	43y/female	Kuwaiti	Kidney transplant, BP, DM	*K*. *pneumoniae*	CRO, MEM	Survived
19	45y/male	Kuwaiti	Kidney transplant, DM, sepsis	*K*. *pneumoniae*	TPZ, MEM	Died from sepsis
20	54y/male	Kuwaiti	Kidney transplant, DM, IHD	*K*. *pneumoniae*	TZP, MEM, CAZ	Survived
21	43y/male	Kuwaiti	Kidney transplant, DM, polycystic kidney disease	*K*. *pneumoniae*	MEM, CTX, CIP	Survived

DM = Diabetes mellitus; BP = hypertension; CVA = cerebrovascular accident; CKD = chronic kidney disease; IHD = ischemic heart disease; DVT = deep venous thrombosis; PVD = peripheral vascular disease; IHD = ischemic heart disease; CML = chronic myeloid leukemia; SLE = systemic lupus erythematosus; TZP = piperacillin-tazobactam; CLAR = clarithromycin; AMC = amoxicillin-clavulanic acid; CIP = ciprofloxacin; NIT = nitrofurantoin; CST = colistin; FEP = cefepime; MTZ = metronidazole; MEM = meropenem; TGC = tigecycline; AMK = amikacin; AMB = amphotericin B; VAN = vancomycin; CRO = ceftriaxone; GEN = gentamicin; CTX = cefotaxime; CAZ = ceftazidime; TEC = teicoplanin; FLC = fluconazole; IPM = imipenem.

As shown in Table 2, the most common infections caused by NDM-1 positive isolates were urinary tract infection (UTI) (n = 10; 47.6%) followed by blood stream infection (BSI) (n = 5; 23.8%), wound/tissue infection (n = 3; 14.3%) and central venous pressure tip infection (n = 1; 4.8%). The remaining 2 patients were colonized as demonstrated by positive rectal and throat screening. All Indian, Syrian, Iranian and Egyptian patients had a recent travel history to their native countries within the previous 1–3 months before admission to the hospital, while only 3 of the Kuwaiti patients had a history of travel to Jordan, India and USA within the previous 1–3 months before hospital admission.

**Table 2:** Source, travel history and ESBL-encoding gene co-harbored by NDM-1 producing *Enterobacteriaceae* isolates.

Patient number (organism)	Specimen source	Associated β-lactamases	History of travel	Co-harbored MBL gene(s)
1 (*E*. *coli*)	Urine	CMY-4, CTX-M-27	None	None
2 (*M*. *morganii*)	Urine	None	India	None
3 (*K*. *pneumoniae*)	Blood	CTX-M-15, SHV-1	Egypt	None
4 (*E*. *coli*))	Urine	CTX-M-15, CMY-4	India	None
5 (*K*. *pneumoniae*)	Wound	CTX-M-15, SHV-1	None	None
6 (*K*. *pneumoniae*)	Urine	CTX-M-15, SHV-11	Jordan	None
7 (*P*. *stuartii*)	Urine	CTX-M-15	None	None
8 (*K*. *pneumoniae*)	Urine	None	None	None
9 (*E*. *coli*)	Screening rectal swab	None	India	None
10 (*Enterobacter cloacae*)	Urine	CMY-4	Syria	None
11(*K*. *pneumoniae*)	Urine	CTX-M-15	None	OXA-48
12(*K*. *pneumoniae*)	Blood	CTX-M15	None	OXA-48
13(*K*. *pneumoniae*)	Urine	CTX-M-15, SHV-1	USA	KPC, OXA-48
14(*K*. *pneumoniae*)	Blood	CTX-M-15, SHV-11	None	KPC, OXA-48
15(*K*. *pneumoniae*)	Screening throat swab	CTX-M-15, SHV-11	Iran	KPC, OXA-48
16 (*E*. *coli*)	Urine	CTX-M-1	None	None
17(*K*. *pneumoniae*)	Blood	CTX-M-1	India	None
18(*K*. *pneumoniae*)	Wound	CMY-4	None	KPC, OXA-48
19(*K*. *pneumoniae*)	Blood	CMY-4	None	KPC, OXA-48
20(*K*. *pneumoniae*)	Tissue	CMY-4	None	KPC, OXA-48
21(*K*. *pneumoniae*)	CVP tip	None	None	KPC, OXA-48

CVP = Central venous pressure

### Susceptibility testing

The results of susceptibility testing are shown in Table 3. All NDM-1 positive isolates were highly resistant to ampicillin, amoxicillin-clavulanic acid, cephalothin, cefotaxime, cefoxitin, meropenem and ertapenem. All except one isolates were resistant to cefepime, imipenem and piperacillin-tazobactam. Nineteen (90.5%), 19 (90.5%) and 14 (66.6%) isolates were resistant to aztreonam, ciprofloxacin and amikacin, respectively. Approximately 50% of isolates were resistant to tigecycline. Two *K*. *pneumoniae* isolates were resistant to colistin with an MIC of 4 and 48mg/L respectively, while the *M*. *morganii* and *P*. *stuartii* isolates were resistant to colistin.

**Table 3:** Antibiotic susceptibility profile of NDM-1-producing *Enterobacteriaceae* with breakpoints (mg/L).

Patient number (organism)	AMK (16)	ATM (4)	FEP (8)	FOX (8)	CTX (1)	TZP (4)	CIP (1)	CST (2)	ETP (0.5)	GEN (4)	IPM (1)	MEM (1)	TGC (2)
1 (*E*. *coli*)	256	24	64	256	256	256	32	0.5	8	1024	32	32	1
2(*M*. *morgannii*)	256	256	256	256	256	256	32	256	4	1024	16	12	4
3(*K*. *pneumoniae*)	24	24	256	256	256	256	32	.125	32	0.125	32	32	0.75
4 (*E*. *coli*)	256	256	96	256	256	256	>32	0.125	>32	1024	1.5	32	0.25
5(*K*. *pneumoniae*)	256	256	256	256	256	256	>32	0.38	>32	512	32	32	1.5
6(*K*. *pneumoniae*)	4	64	256	256	256	256	1	0.75	>32	2	>32	>32	1
7 (*P*. *stuartii*)	1.5	0.25	0.25	256	256	1.5	1.5	256	3	0.125	1	1.5	2
8(*K*. *pneumoniae*)	16	96	256	256	256	256	0.19	0.38	16	1	32	6	0.75
9 (*E*. *coli*)	3	0.064	256	256	256	256	>32	0.25	>32	24	4	12	0.5
10 (*Enterobacter cloacae*)	>256	128	32	256	256	256	12	0.38	>32	1024	32	32	3
11(*K*. *pneumoniae*)	16	128	64	256	256	256	32	4	>32	3	>32	24	16
12(*K*. *pneumoniae*)	16	96	256	256	256	256	32	0.75	32	3	>32	>32	8
13(*K*. *pneumoniae*)	256	256	128	256	256	256	16	0.25	>32	512	>32	>32	0.5
14(*K*. *pneumoniae*)	128	128	256	256	256	256	32	0.38	>32	64	>32	>32	4
15(*K*. *pneumoniae*)	16	128	256	256	256	256	16	0.25	>32	64	16	16	0.5
16 (*E*. *coli*)	128	256	256	256	256	256	32	0.38	>32	64	>32	>32	8
17(*K*. *pneumoniae*)	32	256	256	256	256	256	32	0.125	>32	0.125	>32	>32	4
18(*K*. *pneumoniae*)	64	128	256	256	256	256	16	0.38	>32	64	>32	>32	0.5
19(*K*. *pneumoniae*)	32	128	256	256	256	256	32	48	>32	128	>32	>32	16
20(*K*. *pneumoniae*)	128	256	256	256	256	256	16	1.5	>32	512	>32	>32	4
21(*K*. *pneumoniae*)	64	128	256	256	256	256	32	0.38	>32	128	>32	>32	2

The values in parenthesis in the first row are the recommended breakpoints for resistance according to CLSI [11]. AMK = amikacin; ATM = aztreonam; FEP = cefepime; FOX = cefoxitin; CTX = cefotaxime; TZP = piperacillin-tazobactam; CIP = ciprofloxacin; CST = colistin; ETP = ertapenem; GEN = gentamicin; IPM = imipenem; MEM = meropenem; TGC = tigecycline.

### Detection of ESBLs and carbapenemases

None of the *bla*_NDM-1_-positive isolates co-harbored *bla*_VIM_, *bla*_IMIP_ or *bla*_GIM_ carbapenemase- mediating genes. Two of the *bla*_NDM-1_-positive isolates co-harbored *bla*_OXA-48_ carbapenemase and 7 co-harbored both *bla*_OXA-48_ and *bla*_KPC_ genes (Table 2). No other carbapenemase genes were detected in our testing panel for the remaining 40 CRE. Most of these *bla*_NDM-1_-carrying CRE also carried other resistant genes conferring ESBLs i.e. CTX-M and SHV. Among the CTX-M variants, there were 10 (47.6%) isolates carrying *bla*_CTX-M-15_, 1 (4.8%) *bla*_CTX-M-27_ and 2 (9.5%) *bla*_CTX-M-1_ groups. Among the SHV variants, there were 3 (14.3%) SHV-11 and 3 (14.3%) SHV-1 groups.

### Clonal relatedness

**Fig 1:**
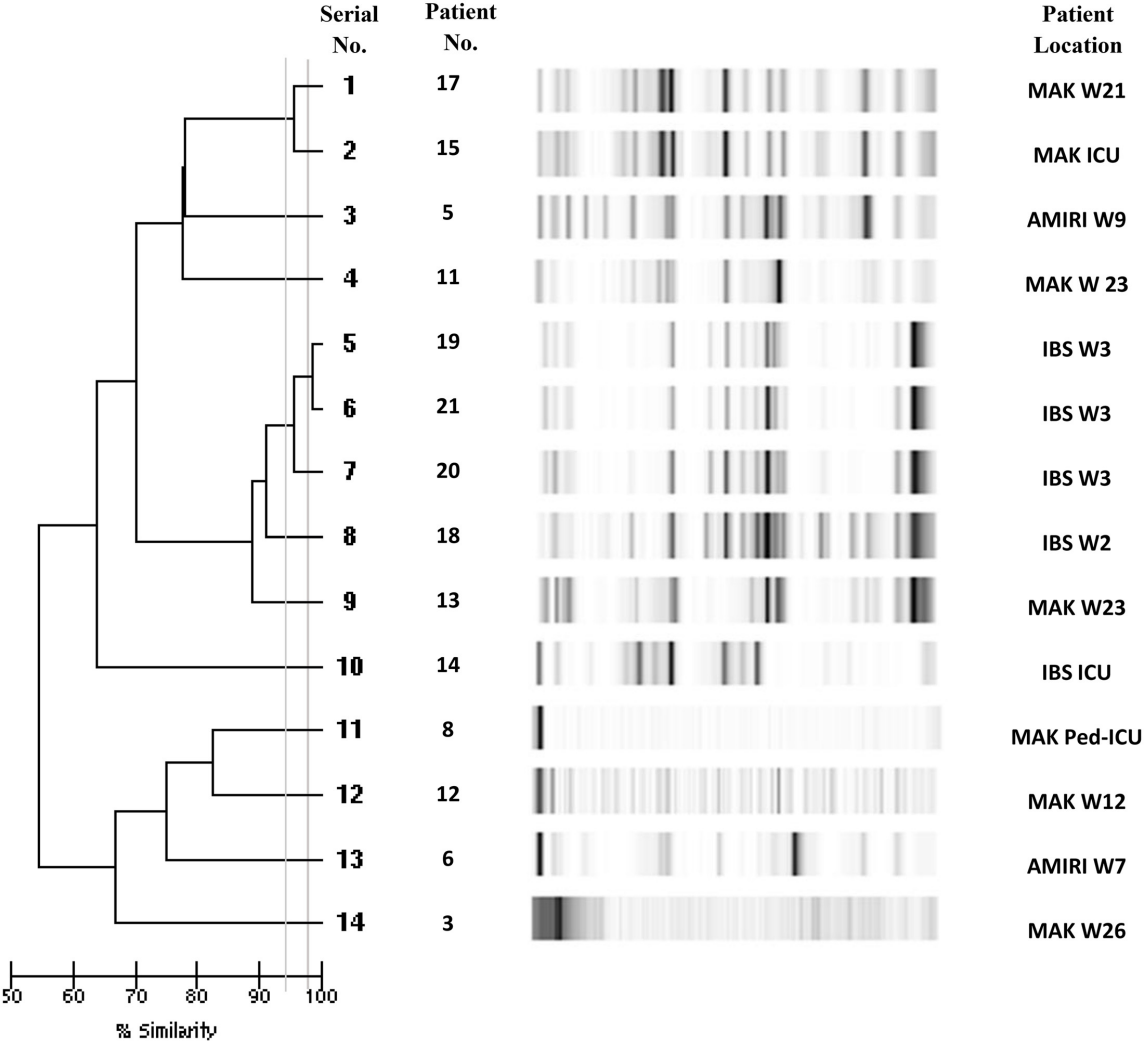
Genetic relatedness analysis of 14 *Klebsiella pneumoniae* isolates with DiversiLab^®^ using repetitive sequence-based polymerase chain reaction (rep-PCR). Dendrogram and computer-generated image of rep-PCR banding patterns are shown. Vertical lines indicate similarities of 95% and 98%. MAK W21 = Mubarak Al Kabeer hospital ward 21; MAK ICU = Mubarak Al Kabeer hospital intensive care unit; Amiri W9 = Amiri hospital ward 9; MAK W23 = Mubarak Al Kabeer hospital ward 23; IBS W3 = Ibn Sina hospital ward 3; IBS W2 = Ibn Sina hospital ward 2; IBS ICU = Ibn Sina hospital intensive care unit; MAK Ped ICU = Mubarak Al Kabeer hospital pediatric intensive care unit; MAK W 12 = Mubarak Al Kabeer hospital ward 12; Amiri W7 = Amiri hospital ward 7; MAK W26 = Mubarak Al Kabeer hospital ward 26

**Fig 2:**
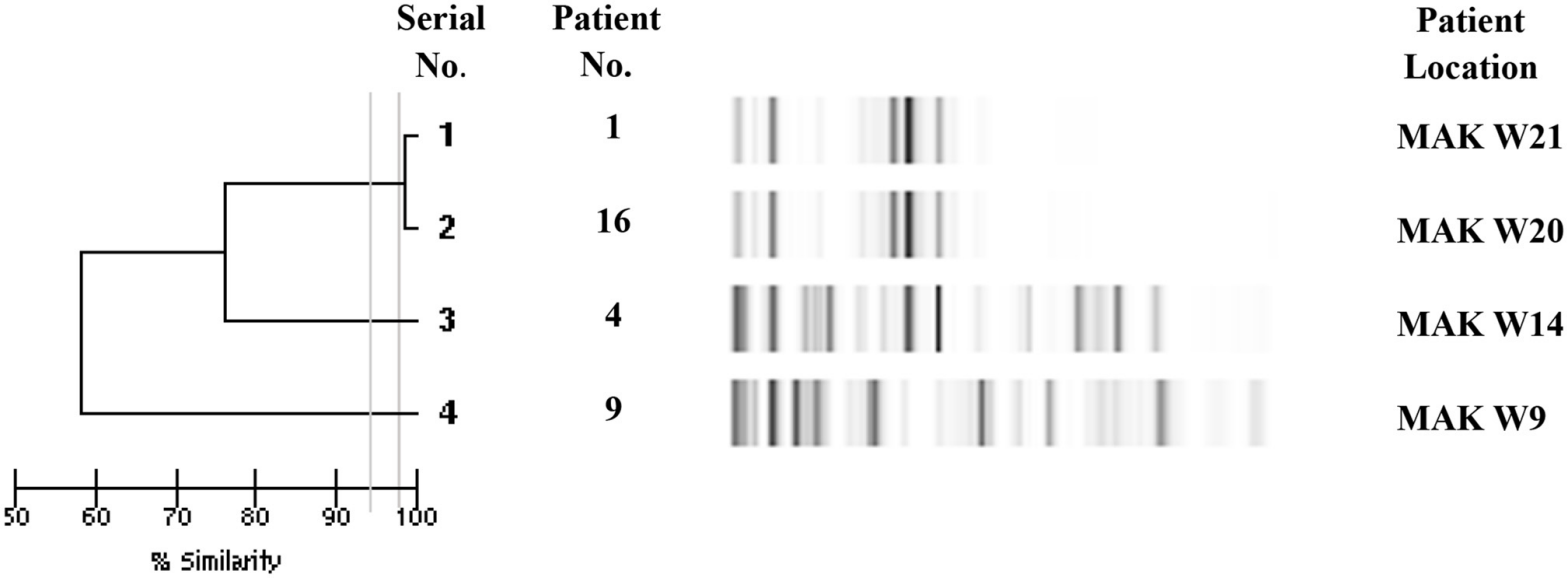
Genetic relatedness analysis of 4 *E*. *coli* strains by DiversiLab^®^ using repetitive sequence-based polymerase chain reaction (rep-PCR). Dendrogram and computer-generated image of rep-PCR banding patterns are shown. Vertical lines indicate similarities of 95% and 98%. MAK W21 = Mubarak Al Kabeer hospital ward; MAK W20 = Mubarak Al Kabeer hospital ward 20; MAK W14 Mubarak Al Kabeer hospital ward 14; MAK W 9 = Mubarak Al Kabeer hospital ward 9

Analysis of genetic relatedness of 14 *K*. *pneumoniae* isolates by Diversilab^®^ revealed a similarity of > 95% for 3 isolates (serial numbers 5, 6, 7; i.e. patients 19, 21 and 20) suggesting a close relationship (Fig 1). However, 2 of these isolates (serial numbers 5 and 6) were ≥ 98% similar suggesting an identity, thus confirming that the same clone infected two patients within one month period in one hospital. In addition, there was > 95% similarity between 2 *K*. *pneumoniae* isolates (serial numbers 1 and 2; i.e. patient 17 and 15). Analysis of genetic relatedness among all 4 *E*. *coli* isolates harboring *bla*_NDM-1_ genes revealed an identity of ≥ 98% for 2 isolates (serial numbers 1, 2; patients 1 and 16), suggesting clonality of these 2 isolates causing infection in two adjacent wards (Fig 2).

## Discussion

The emergence of NDM-1 producing *Enterobacteriaceae* has become a major public health threat that presents a challenge for treatment of serious infections throughout the world. There are several published reports of NDM-1 carbapenemases worldwide including the Gulf Corporation Council (GCC) countries of Saudi Arabia, Sultanate of Oman, Qatar, United Arab Emirates and Kuwait [16, 10], Yemen [17], the West Asian countries of Turkey [18], Lebanon [19] and Israel [20], and North African countries of Egypt [21], Tunisia [22] and Morocco [23]. In our study, 61.9% of the NDM-1 harboring isolates co-expressed ESBLs which is concordant with previous reports [16–19, 23]. The present study reveals the fact that carbapenem resistance among *Enterobacteriaceae* mediated by NDM-1 MBL production is becoming a significant occurrence in several hospitals in Kuwait. NDM-1 was present in 2.7% of all *Enterobacteriaceae* in our study and in about 34.4% of all CRE. This is more than double the 1.2% reported by Kumarasamy and colleagues in 2010 in India, Pakistan and the United Kingdom [24] and the 1.1% reported by Tran and colleagues in Vietnam in 2015 [25]. The detection of 34.4% of the CRE in our hospitals as NDM-1-producers is alarming. However, 40 of the CRE isolates were negative for all the tested β-lactamases and carbapenemases genes, suggesting the possibility of occurrence of new genes or permeability problems [26, 19].

This report reviews 21 cases of infected or colonized patients caused by NDM-1 positive bacteria in Kuwait. They were recognized over a period of one year. The majority of the isolates were from female patients above the age of 53 years of age. Our data are similar to those reported by Kumarasamy *et al*. from India [24], where the majority were female patients but different in the mean ages; 53.3 versus 35years. This is in contrast to the isolates reported from the United Kingdom by Jain and colleagues in 2014, where the majority of their patients were males above 60 years of age [27]. About half of our isolates were from UTI (47.6%), a finding similar to the study of Jain *et al*. [27]. Other frequent sources were blood stream infection (23.8%) and wound/tissue infections (14.3%). History of travel or healthcare contact in endemic areas was given by more than a quarter of our patients. Some patients also travelled to areas regarded as non-endemic as Middle- Eastern countries (Egypt, Syria, Jordan and Iran) and the USA. None-of these patients had a history of travel to the Balkans, which is suggested to be the second epicenter for NDM-positive bacteria [28]. A very high proportion (71.4%) of NDM-1 positive patients had no history of travel to any endemic areas such as India and Pakistan or contact with healthcare facilities which have been reported previously as a source of infection [27, 29]. It appears that all our patients acquired the NDM-1 producing strains while they were in the hospital suggesting nosocomial acquisition. A caveat of this assumption is that the patients were not screened for NDM-1 producing bacteria on the day of hospital admission. Infections due to CRE have been associated with high mortality rates ranging from 38–44% [30, 31]. The mortality rate in our series is slightly lower (28.6%); all died from septic shock.

In this report, all the CRE were resistant to meropenem and ertapenem while 96% of them were resistant to imipenem. Variable resistance to carbapenems has been reported by other workers [19, 32]. This is attributed to alterations in porins leading to variable permeabilities to different carbapenems [33]. Although aztreonam is not inactivated by NDM-1 or other metallo-β-lactamase enzymes, approximately 91% of our CRE were resistant to aztreonam, which is essentially similar to the findings of other studies [24, 27] thus reflecting perhaps the presence of other resistance genes such as ESBLs or AmpC enzymes. Among the non-β-lactam antimicrobial agents, ciprofloxacin resistance was noted in 90.5% of the NDM-1 positive isolates. High-level resistance to amikacin and gentamicin was detected in 66.6% which may be related to the production of one or more of the 16S rRNA methyltransferases by some of the CRE isolates [34]. After exclusion of *Morganella* spp. and *Providentia* spp. which are intrinsically resistant to colistin, 90% of our isolates were susceptible to this drug, a finding already noted in other previous studies [24, 27]. However, after exclusion of *Morganella* spp. and *Providentia* spp. which are intrinsically resistant to tigecycline, only 52.6% of CRE were susceptible to tigecycline, a rate which is higher than those reported previously [24, 27].

There are multiple clones of NDM-1 producing *K*. *pneumoniae* and *E*. *coli* strains circulating in Kuwaiti hospitals. However, two *K*. *pneumoniae* and two *E*. *coli* isolates were clonally identical pointing to the potential of outbreaks due to some clones.

In conclusion, out study has established the prevalence of NDM-1 genes encoding carbapenem resistance amongst *Enterobacteriaceae* isolates in Kuwait and the high frequency of multi-drug resistance among these bacteria. Except for a small outbreak in one hospital, the majority of cases were sporadic. This report highlights the speed at which NDM-1-mediated resistance is spreading in the country which was not the case three years ago, when there was no case of CRE and MBL-positive isolates. This spread appears to be facilitated by different factors such as immigration, poor infection control practices, antibiotic misuse, and environmental spread.

Monitoring and control measures are important to prevent the spread of CRE. The Centers for Disease Control (CDC), Atlanta, GA, USA have published the guidelines for these [35]. The measures include: hand hygiene, contact precautions, healthcare personnel education, minimal use of invasive devices, laboratory reporting of CRE isolation to clinical and infection control staff, identification of CRE patients at admission, antibiotic stewardship, environmental cleaning, patient and staff cohorting, screening contacts of CRE patients, active surveillance testing and chlorhexidine bathing.

## References

[1] Giske CG, Monnet DL, Cars O, Carmeli Y, ReAct-Action on antibiotic resistance. Clinical and economic impact of common multidrug-resistant gram-negative bacilli. Antimicrob Agents Chemother. 2008; 52: 813–821. 18070961 10.1128/AAC.01169-07 PMC2258516

[2] Nordmann P, Naas T, Poirel L. Global spread of carbapenemase-producing *Enterobacteriaceae*. Emerg Infect Dis. 2011; 17: 1791–1798. 10.3201/eid1710.110655 22000347 PMC3310682

[3] Centers for Disease Control and Prevention (CDC). Antibiotic resistance threats in the United States. Atlanta, GA, USA 2013 Available: http://www.cdc.gov/drugresistance/threat-report-2013/.

[4] Yong D, Toleman MA, Giske CG, Cho HS, Sundman K, Lee K, et al Characterization of a new metallo-β-lactamase-1 gene, *bla*_NDM-1_, and a novel erythromycin esterase gene carried on a unique genetic structure in *Klebsiella pneumoniae* sequence type 14 from India. Antimicrob Agents Chemother. 2009; 53: 5046–5054. 10.1128/AAC.00774-09 19770275 PMC2786356

[5] Johnson AP, Woodford N. Global spread of antibiotic resistance: the example of New Delhi metallo-β-lactamase (NDM)–mediated carbapenem resistance. J Med Microbiol. 2013; 62: 499–513. 10.1099/jmm.0.052555-0 23329317

[6] Moellering RC Jr. NDM-1- a cause for worldwide concern. N Engl J Med. 2010; 363: 2377–2379. 10.1056/NEJMp1011715 21158655

[7] Al Sweih N, Jamal W, Rotimi VO. Spectrum and antibiotic resistance of uropathogens isolated from hospital and community patients with urinary tract infections in two large hospitals in Kuwait. Med Princ Pract. 2005; 14: 401–407. 16220013 10.1159/000088113

[8] Foxman B. Urinary tract infection syndromes: occurrence, recurrence, bacteriology, risk factors, and disease burden. Infect Dis Clin North Am. 2014; 28: 1–13. 10.1016/j.idc.2013.09.003 24484571

[9] Jamal W, Rotimi VO, Albert MJ, Khodakhast F, Udo EE, Poirel L. Emergence of nosocomial New Delhi metallo-β-lactamase-1 (NDM-1)-producing *Klebsiella pneumoniae* in patients admitted to a tertiary care hospital in Kuwait. Int J Antimicrob Agents. 2012; 39: 183–184. 10.1016/j.ijantimicag.2011.10.002 22113192

[10] Jamal W, Rotimi VO, Albert MJ, Khodakhast F, Nordmann P, Poirel L. High Prevalence of VIM-4 and NDM-1 metallo-β-lactamase among carbapenem–resistant *Enterobacteriaceae*. J Med Microbiol. 2013; 62: 1239–1244. 10.1099/jmm.0.059915-0 23639985

[11] Clinical and Laboratory Standards Institute (CLSI). Performance Standards for Antimicrobial susceptibility testing; Twenty Fourth Informational Supplement M100-S24. Wayne, Philadelphia; 2014.

[12] Rotimi VO, Jamal W, Pal T, Sovenned A, Albert MJ. Emergence of CTX-M-15 type extended-spectrum β-lactamase–producing *Salmonella* spp. in Kuwait and the United Arab Emirates. J Med Microbiol. 2008; 57: 881–886. 10.1099/jmm.0.47509-0 18566147

[13] Poirel L, Walsh TR, Cuvillier V, Nordmann P. Multiplex PCR for detection of acquired carbapenemase genes. Diagn Microbiol Infect Dis. 2011; 70: 119–123. 10.1016/j.diagmicrobio.2010.12.002 21398074

[14] Lukinmaa-Aberg S, Horsma J, Pasanen T, Mero S, Aulu L, Vaara M, et al Application of DiversiLab repetitive sequence-based PCR method in epidemiological typing of Enterohemorrhagic *Escherichia coli* (EHEC). Foodborne Pathog Dis. 2013; 10:632–638. 10.1089/fpd.2012.1411 23692078

[15] Fluit AC, Terlingen AM, Andriessen L, Ikawaty R, van Mansfeld R, Top J, et al Evaluation of the DiversiLab system for detection of hospital outbreaks of infections by different bacterial species. J Clin Microbiol. 2010; 48: 3979–3989. 10.1128/JCM.01191-10 20861340 PMC3020881

[16] Zowawi HM, Sartor AL, Balkhy HH, Walsh TR, Al Johani SM, AlJindan RY, et al Molecular characterization of carbapenemase-producing *Escherichia coli* and *Klebsiella pneumoniae* in the countries of the Gulf Cooperation Council: dominance of OXA-48 and NDM producers. Antimicrob Agents Chemother. 2014; 58: 3085–3090. 10.1128/AAC.02050-13 24637692 PMC4068443

[17] Gharout-Sait A, Alsharapy S-A, Brasme L, Touati A, Kermas R, Bakour S, et al *Enterobacteriaceae* isolates carrying the New Delhi mettalo-β-lactamase gene in Yemen. J Med Microbiol. 2014; 63: 1316–1323. 10.1099/jmm.0.073767-0 25009193

[18] Poirel L, Yilmaz M, Istanbullu A, Arslan F, Mert A, Bernabeu S, et al Spread of NDM-1-producing *Enterobacteriaceae* in a neonatal intensive care unit in Istanbul, Turkey. Antimicrob Agents Chemother. 2014; 58: 2929–2933. 10.1128/AAC.02047-13 24550328 PMC3993247

[19] Baroud M, Dandache I, Araj GF, Wakim R, Kanji S, Kanafani Z, et al Underlying mechanisms of carbapenem resistance in extended-spectrum β-lactamase-producing *Klebsiella pneumoniae* and *Escherichia coli* isolates at a tertiary care centre in Lebanon: role of OXA-48 and NDM-1 carbapenemases. Int J Antimicrob Agents. 2013; 41: 75–79. 10.1016/j.ijantimicag.2012.08.010 23142087

[20] Lachish T, Elimelech M, Arieli N, Adler A, Rolain JM, Assous MV. Emergence of New Delhi metallo-β-lactamase in Jerusalem, Israel. Int J Antimicrob Agents. 2012; 40: 566–567. 10.1016/j.ijantimicag.2012.07.011 22951226

[21] Bathoorn E, Friedrich AW, Zhou K, Arends JP, Borst DM, Grundmann H, et al Latent induction to the Netherlands of multiple antibiotic resistance including NDM-1 after hospitalization in Egypt, August 2013. Euro surveill. 2013; 18 10.2807/1560-7917.ES2013.18.42.20610 24176580

[22] Ben Nasr A, Decre D, Compain F, Genel N, Barguellil F, Arlet G. Emergence of NDM-1 in association with OXA-48 in *Klebsiella pneumoniae* from Tunisia. Antimicrob Agents Chemother. 2013; 57: 4089–4090. 10.1128/AAC.00536-13 23752514 PMC3719753

[23] Poirel L, Benouda A, Hays C, Nordmann P. Emergence of NDM-1-producing *Klebsiella pneumonie* in Morocco. J Antimicrob Chemother. 2011; 66: 2781–2783. 10.1093/jac/dkr384 21930570

[24] Kumarasamy KK, Toleman MA, Walsh TR, Bagaria J, Butt F, Balakrishnan R, et al Emergence of a new antibiotic resistance mechanism in India, Pakistan, and the UK: a molecular, biological, and epidemiological study. Lancet Infect Dis. 2010; 10: 597–602. 10.1016/S1473-3099(10)70143-2 20705517 PMC2933358

[25] Tran HH, Ehsani S, Shibayama K, Matsui M, Suzuki S, Nguyen MB, et al Common isolation of New Delhi Metallo-beta-lactamase-1-producing *Enterobacteriaceae* in a large surgical hospital in Vietnam. Eur J Clin Microbiol Infect Dis. 2015; 34: 1247–1254. 10.1007/s10096-015-2345-6 25732142 PMC4426131

[26] Dortet L, Poirel L, Abbas S, Oueslati S, Nordmann P. Genetic and biochemical characterization of FRI-1, a carbapenem-hydrolyzing class A β-lactamase from *Enterobacter cloacae*. Antimicrob Agents Chemother. 2015; 59: 7420–4725. 10.1128/AAC.01636-15 26392482 PMC4649213

[27] Jain A, Hopkins KL, Turton J, Doumith M, Hill R, Loy R, et al NDM carbapenemases in the United Kingdom: an analysis of the first 250 cases. J Antimicrob Chemother. 2014; 69: 1777–1784. 10.1093/jac/dku084 24769387

[28] Struelens MJ, Monnet DL, Magiorakos AP, Santos O’Connor F, Giesecke J, the European NDM-1 survey participants. New Delhi metallo-β-lactamase 1-producing *Enterobacteriaceae*: emergence and response in Europe. Euro Surveill. 2010; 15 Available: http://www.eurosurveillance.org/ViewArticle.aspx?articleld=19716. 10.2807/ese.15.46.19716-en 21144431

[29] Nordmann P, Couard J-P, Sansot D, Poirel L. Emergence of an autochthonous and community–acquired NDM-1-producing *Klebsiella pneumoniae* in Europe. Clin Infect Dis. 2012; 54: 150–151. 10.1093/cid/cir720 21960718

[30] Patel G, Huprikar S, Factor SH, Jenkins SG, Calfee DP. Outcomes of carbapenem-resistant *Klebsiella pneumoniae* infection and the impact of antimicrobial and adjunctive therapies. Infect Control Hosp Epidemiol. 2008; 29: 1099–1106. 10.1086/592412 18973455

[31] Schwaber MJ, Klarfeld-Lidji S, Navon-Venezia S, Schwartz D, Leavitt A, Carmeli Y. Predictors of carbapenem–resistant *Klebsiella pneumoniae* acquisition among hospitalized adults and effect of acquisition on mortality. Antimicrob Agents Chemother. 2008; 52: 1028–1033. 18086836 10.1128/AAC.01020-07 PMC2258527

[32] Osterblad M, Kirveskari J, Hakanen AJ, Tissari P, Vaara M, Jalava J. Carbapenemase –producing Enterobacteriaceae in Finland: the first years (2008–11). J Antimicrob Chemother. 2012; 67: 2860–2864. 10.1093/jac/dks299 22855858

[33] Garcia-Fernandez A, Miriagou V, Papagiannitsis, Giordano A, Venditti M, Mancini C, et al An ertapenem-resistant extended-spectrum-β-lactamase-producing *Klebsiella pneumoniae* clone carries a novel OmpK36 porin variant. Antimicrob Agents Chemother. 2010; 54: 4178–4184. 10.1128/AAC.01301-09 20660683 PMC2944588

[34] Hidalgo L, Hopkins KL, Gutierrez B, Ovejero CM, Shukla S, Douthwaite S, et al Association of the novel aminoglycoside resistance determinant RmtF with NDM carbapenemase in *Enterobacteriaceae* isolated in India and the UK. J Antimicrob Chemother. 2013; 68: 1543–1550. 10.1093/jac/dkt078 23580560

[35] Centers for Disease Control and Prevention (CDC). Facility guidance for control of carbapenem-resistant *Enterobacteriaceae* (CRE). November 2015 update- CRE toolkit. Available: http://www.cdc.gov/hai/pdfs/cre/CRE-guidance-508/.

